# When Electrospun Fiber Support Matters: In Vitro Ovine Long-Term Folliculogenesis on Poly (Epsilon Caprolactone) (PCL)-Patterned Fibers

**DOI:** 10.3390/cells11121968

**Published:** 2022-06-19

**Authors:** Chiara Di Berardino, Liliana Liverani, Alessia Peserico, Giulia Capacchietti, Valentina Russo, Nicola Bernabò, Umberto Tosi, Aldo Roberto Boccaccini, Barbara Barboni

**Affiliations:** 1 Faculty of Bioscience and Technology for Food, Agriculture and Environment, University of Teramo, 64100 Teramo, Italy; apeserico@unite.it (A.P.); gcapacchietti@unite.it (G.C.); vrusso@unite.it (V.R.); nbernabo@unite.it (N.B.); utosi@unite.it (U.T.); bbarboni@unite.it (B.B.); 2 Institute of Biomaterials, Department of Materials Science and Engineering, Friedrich-Alexander University of Erlangen-Nuremberg, 91054 Erlangen, Germany; liliana.liverani@fau.de (L.L.); aldo.boccaccini@fau.de (A.R.B.)

**Keywords:** artificial-ovary, PCL-electrospun scaffold, patterned fibers, random fibers, in vitro follicle culture, preantral follicle growth, oocyte meiotic competence, sheep

## Abstract

Current assisted reproduction technologies (ART) are insufficient to cover the slice of the population needing to restore fertility, as well as to amplify the reproductive performance of domestic animals or endangered species. The design of dedicated reproductive scaffolds has opened the possibility to better recapitulate the reproductive 3D ovarian environment, thus potentially innovating in vitro folliculogenesis (ivF) techniques. To this aim, the present research has been designed to compare ovine preantral follicles in vitro culture on poly(epsilon-caprolactone) (PCL)-based electrospun scaffolds designed with different topology (Random vs. Patterned fibers) with a previously validated system. The ivF performances were assessed after 14 days under 3D-oil, Two-Step (7 days in 3D-oil and on scaffold), or One-Step PCL protocols (14 days on PCL-scaffold) by assessing morphological and functional outcomes. The results show that Two- and One-Step PCL ivF protocols, when performed on patterned scaffolds, were both able to support follicle growth, antrum formation, and the upregulation of follicle marker genes leading to a greater oocyte meiotic competence than in the 3D-oil system. In conclusion, the One-Step approach could be proposed as a practical and valid strategy to support a synergic follicle-oocyte in vitro development, providing an innovative tool to enhance the availability of matured gametes on an individual basis for ART purposes.

## 1. Introduction

Established Assisted Reproductive Technologies (ART) approaches remain insufficient to preserve reproductive function in cases of the premature reduction in female gamete reserve, such as in pre-pubertal cancer patients [1], as well as in premature ovarian failure or in gonadotoxic treatment [2,3], or to amplify the reproductive performance in animals. In the latter instance, any new technology aimed to enhance the reproductive performance of highly-selected domestic mammals or to preserve biodiversity by contrasting the reduced numerical strength of mammalian endangered species remain a challenging target of ART [4,5].

The transplantation of cryopreservation of ovarian tissue is, to date, considered a valid strategy to preserve or extend female fertility [6,7,8,9,10,11,12], even if this treatment may be unsafe in cancer patients since it exposes them to the potential risk of malignant cells reintroduction [1]. A technological alternative to reach a similar clinical target is the possibility of recruiting a large reserve of immature female gametes through in vitro folliculogenesis (ivF) [13]. This emerging technology may potentially offer the opportunity to reproduce in culture the phase of follicle and oocyte growth by obtaining fully-grown meiotically competent oocytes to address in vitro maturation/fertilization (IVM/IVF) and embryo culture protocols [2,3,14].

In particular, ivF of single preantral (PA) follicles or of slices of the ovarian cortex [15,16,17] have been proposed in different mammalian models [14,18,19,20,21], reaching, however, a modest degree of efficacy [22].

To overcome these claims, the reproductive research and innovation sector pays great attention to the emerging cutting-edge strategy based on the use of a 3D artificial environment [1,23,24,25]. These innovative approaches potentially enable the increasingly physiological in vitro modelling of reproductive key events. The rationale of 3D technologies consists of engineering female biomimetic reproductive tissues, thus generating 3D integrated multi-organ in vitro systems in which the different cell compartments that physiologically control female reproductive homeostasis and function may cooperate in generating the complexity of the endocrine and paracrine mechanisms controlling reproductive outcomes.

Currently, the reproductive biotechnology community is growing the attention to tissue engineering advances [26,27] by taking advantage of the synergetic convergence of the advanced knowledge of reproductive biology developed to date with the technological dynamism of the sector, applied to the fabrication of customized biomaterials and native matrixes [28,29] essential to innovate both protocols the in vivo ovarian transplantation and ivF, thus providing new opportunities in the “avant-garde” of ART.

In particular, 3D ARTs are based on the availability of tissue-specific scaffolds, that have to be engineered to recapitulate a 3D reproductive environment (REPROTEN: REPROductive Tissue ENgineering) [28].

The bioengineered artificial ovary could be able to mimic the ovarian native system, which is why an appropriate biocompatible scaffold to encapsulate the isolated follicles and autologous ovarian cells is required, whose co-presence may be relevant for the survival and follicular development [13]. Several studies report live births from biomaterial implants in mice using isolated follicles or whole-ovarian tissue [30,31,32]. Nevertheless, when translating this work to a large animal or human [25], the implant must house a significantly larger follicles pool, considerably larger than those used in mice.

These technologies, although holding enormous potential, remain at the preclinical stage and are primarily a proof of concept due to the complexity of recapitulating the in vivo ovarian environment [25]. However, scaffolds fulfilling these biological goals have been fabricated and investigated, and they are mainly composed of natural and synthetic polymers, for example, collagen, fibrin, plasma clots, alginate, and decellularized ovarian ECM [25]. Among the scaffold fabrication techniques, 3D printing and electrospinning have been investigated for the fabrication of constructs able to support the growth and development of ovarian follicles [25,33,34,35]. Even if several biomaterials could be potentially applied to this purpose, only the use of a few of them, such as gelatin (mildly crosslinked), poly(epsilon-caprolactone), and its blend with gelatin, has been reported.

The wide relevance of the REPROTEN-related material sector has been applied to electrospinning, a widely used technique for the fabrication of scaffolds that are able to mimic the nanofibrillary structure of the native tissue ECM. Among these scaffolds, the poly(epsilon-caprolactone) (PCL)-based scaffolds are noteworthy [36]. In fact, PCL is a biodegradable polymer that has been successfully used in vivo and in vitro for regeneration purposes in other tissues such as bone [37,38,39,40,41], cartilage [42,43,44,45,46], osteochondral [47,48,49,50,51,52], skin [53,54,55,56,57], nerve [58,59,60,61], cardiovascular [62,63], musculoskeletal [64,65,66], liver [67,68,69], and dental [70,71,72,73,74,75,76] tissue regeneration.

Recently, PCL electrospun scaffold technology has also been applied to the fabrication of engineered reproductive materials, even if, at the moment, only a few groups of researchers are focusing the activities on this topic, representing a relevant novelty in the field [33,34,77].

In this context, recent studies have reported the sustainability of poly (epsilon-caprolactone) (PCL)-based electrospun patterned scaffolds as support for ovarian follicles growth, viability, and preservation of the fibrillary morphology of the native follicular unit [33,34,77,78]. Although PCL-based scaffolds have been found to be able to promote porcine follicle survival, thus supporting the proof of concept of its use to generate artificial ovaries for transplantation; however, no information has been collected to date on the ability of PCL electrospun REPROTEN scaffolds in supporting in vitro follicle/oocyte development.

To this aim, the present research has been designed in order to compare a validated 3D-oil ivF system developed for PA follicles [79] with 3D transwell culture protocols performed by using holders filled with PCL electrospun porous scaffolds polymer with randomic or patterned fibers topology.

The performance of different ivF culture conditions was assessed by detecting the development of somatic and germinal compartments after 14 days of follicle incubation by combining morphological (follicle/oocyte growth, timing, and the percentage of antrum differentiation) and functional data (gene expression in granulosa cell and acquisition of meiotic competence for oocyte).

The optimization of the 3D PA culture system was carried out using the ovine model by considering the pivotal role that this medium-sized ruminant model plays in the reproductive research context [16,20,80,81,82,83,84,85] and its high translational value [86].

## 2. Materials and Methods

### 2.1. Chemicals

All of the chemicals used in this study were purchased from Sigma (Sigma Chemical Co., St. Louis, MO, USA) unless otherwise indicated.

### 2.2. Scaffold Fabrication

#### Poly(Epsilon-Caprolactone) (PCL) Patterned Electrospun Scaffolds/(PCL) Randomic Electrospun Scaffolds

The PCL electrospun scaffolds were fabricated according to the protocols reported in our previous works [33,78]. Briefly, the patterned and randomly oriented scaffolds were obtained by using the same polymeric solution. The solution for the electrospinning was prepared by dissolving PCL (average Mn 80,000) in glacial acetic acid (VWR, Darmstadt, Germany), with a concentration of 20% *w/v*, stirred overnight, and electrospun directly after immersion for 1 h in an ultrasound bath. The process parameters used for both scaffold types were kept constant, except for the use of a dedicated collector for the pattern. In detail, the applied voltage was set at 15 kV, the distance between the tip of the needle and the collector was 11 cm, the flow rate of the solution was 0.4 mL/h, and the needle diameter was 23 G. The used electrospinning device was equipped with a climate chamber, which allowed the setting and control of the temperature and relative humidity during the process (EC-CLI, IME Medical Electrospinning, Geldrop, The Netherlands).

The electrospun fiber morphology and scaffold topology were investigated by using a scanning electron microscope (SEM) (Auriga Base, Zeiss, Jena, Germany).

### 2.3. Isolation, Morphological Evaluation, and In Vitro Culture of PA Follicles

The present research was designed to perform in vitro follicle growth of preantral follicles (PA) isolated from the Appenninica breed of sheep lamb ovaries collected at the slaughterhouse from animals intended for consumption [86]. Twelve ewes about 5 months old and a total of 24 ovaries were used for this purpose.

The ovaries were transported to the laboratory in a thermostatic container (38 °C during transportation from the slaughterhouse to the laboratory) within 1 h, rinsed in NaCl 0.9% solution supplemented with Benzoxonium chloride 1 mg/mL (Cat. No. 032186013 Bialcol Med, Vemedia Pharma S.r.l., Parma, Italy) before manipulation.

After the removal of the medulla, the ovaries were transferred into HEPES-buffered TCM199 medium (Cat. No. M7528 Sigma) and cut into cortical strips (0.5 × 0.5 × 0.5 cm). PA follicles were mechanically isolated from cortical strips with 32 G sterile needles under the stereomicroscope in the flow hood to avoid theca layer damage, and they were selected on the basis of their morphology and size [21]. The medium-large PA follicles used for the in vitro culture experiments showed a mean diameter of 250.5 ± 4 μm. This category of PA follicles showing the greatest in vitro growing performance [18,21,79] was identified under an inverted-phase microscope associated with the time-lapse imaging software NIS-Elements (Eclipse Ti Series, Nikon, Tokyo, Japan) to identify the PA diameter. Furthermore, this selection allowed for the exclusion of follicles with damaged basal membranes, and the follicles with early signs of degeneration were discarded (follicles without follicular 3D microarchitecture, presenting with extrusion of the oocytes, and displaying a darkness aspect of the somatic compartment). The culture was carried out at 38.5 °C and 5% CO_2_ for 14 days in MEM alpha (aMEM; Cat. No. BE02-002F Lonza, Basel, Switzerland) with 5% Knockout™ Serum Replacement (Knockout™ SR; Cat. No. 10828028 Gibco, Thermo Fisher Scientific, Waltham, MA, USA), 1% ITS (insulin, transferrin, and selenium; Cat. No. I1884 Sigma), 50 μg/mL of ascorbic acid (Cat. No. A4544 Sigma), 2 mM of glutamine (Cat. No. BE17-605E/U1 Lonza), and antibiotics (75 mg/L of penicillin-G, 50 mg/L of streptomycin sulfate; Cat. No. DE17-602E Lonza) [86]. The culture medium was changed every 48 h and supplemented with 4 IU/mL of equine Chorionic Gonadotropin (eCG; corresponding to 1 μg/mL FOLLIGON^®^, MSD Animal Health S.r.l., Segrate, Italy), as previously validated [86]. The eCG biological activity was declared to be 5000 IU per vial according to the manufacturer’s instructions.

### 2.4. In Vitro PA Follicles Culture Protocols Comparison

**Figure 1:**
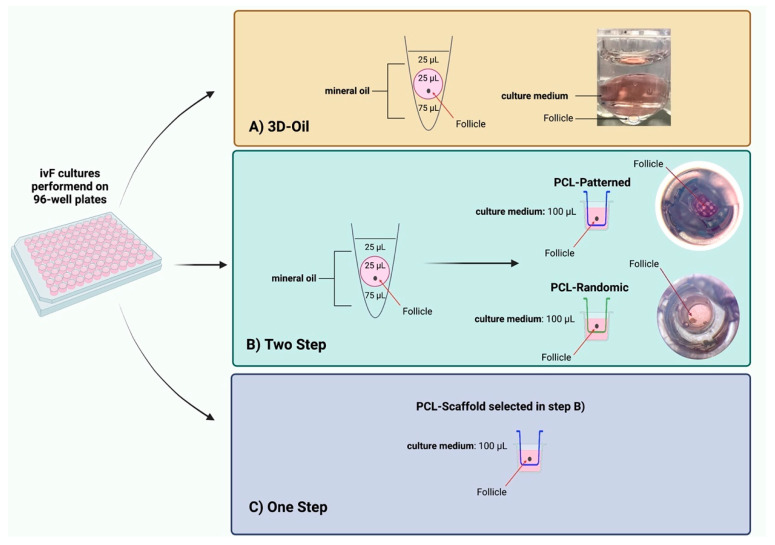
ivF culture protocols carried out on single follicle. Two-Step culture protocols with PCL-Randomic and PCL-Patterned fibrous scaffold were compared to the previously validated 3D-oil protocol. Secondly, Two-Step and One-Step PCL-Patterned approaches performances were compared. Specifically: (**A**) in 3D-oil, single PA follicles were placed in a 25 μL drop of culture medium under a 75 μL drop of pre-equilibrated mineral oil (density 5 0.84 g/mL) and then overlaid by 25 μL oil in a 96-well dish with V-shaped wells; (**B**) in Two-Step, single PA follicles were placed in a 25 μL drop of culture medium under a 75 μL drop of pre-equilibrated mineral oil (density 5 0.84 g/mL) and then overlaid by 25 μL oil in a 96-well dish with V-shaped wells before antrum formation (day 6 of ivF culture) to then move the growing single follicle on trans-well culture system with the holder filled with PCL-Randomic or Patterned electrospun scaffolds (PCL-Randomic vs. PCL-Patterned); (**C**) in One-Step, single PA follicles were in vitro cultured on the trans-well culture system with one of the electrospun PCL-scaffold identify during the (**B**) step for 14 days. Three independent biological replicates were performed, and for each protocol a total of 120, 125, 122, and 120 follicles were considered (respectively for 3D-oil, Two-Step PCL-Randomic, Two-Step PCL-Patterned, One-Step PCL-Patterned), and compared the ivF performances by assessing follicular (follicles in vitro development and granulosa cells’ gene expression) and germinal (acquisition of meiotic competence using IVM) compartments. Created with BioRender.com.

The healthy medium-large PA follicles were randomly assigned to different culture ivF protocols, and the procedure was carried out by adopting single-follicle approaches (Figure 1): (A) 3D-oil validated method [14,79,86]; (B) Two-Step protocol designed by starting ivF culture, before antrum formation, on the 3D-oil system (day 6 of ivF culture) to then move the growing single follicle onto the trans-well culture system with the holder filled with PCL-Randomic or Patterned electrospun scaffolds (PCL-Randomic vs. PCL-Patterned); (C) One-Step protocol designed by carrying out whole the PA follicle culture (14 days) on the trans-well culture system with one of the electrospun PCL-scaffold identify during the B) step (Figure 1). Three independent biological replicates were performed for each tested protocol, comparing the performances of in vitro folliculogenesis by assessing the morphological and functional analysis of the in vitro follicle development and meiotic competence acquisition of in vitro follicle-grown oocytes.

### 2.5. In Vitro PA Follicles Culture Outcomes

#### 2.5.1. Morphological and Functional Analysis of In Vitro Follicle Development

The ivF outcomes at the end of the culture (14 days) were defined by considering follicle growth, antral cavity differentiation, and the incidence of degeneration.

The in vitro follicle growth was analyzed by recording the final diameters detected using an inverted microscope (time-lapse imaging software NIS-Elements (Eclipse Ti Series, Nikon, Japan) and expressed as the rate of increasing size (∆ growth %).

**Figure 2:**
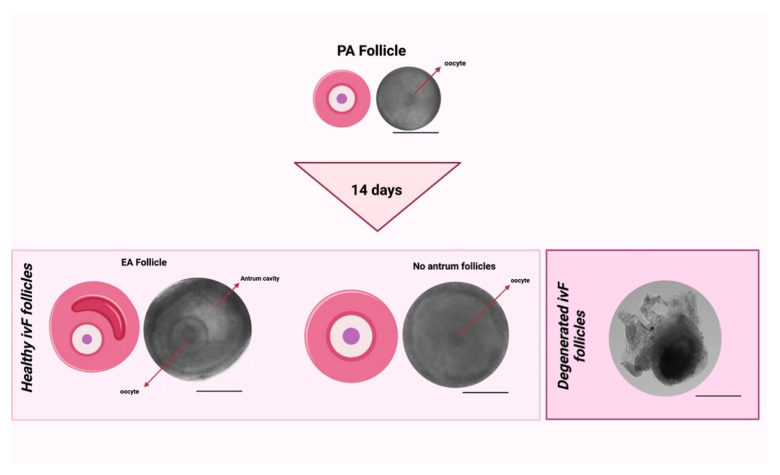
Comparison between healthy follicles after ivF cultures (no antrum and EA follicles) vs. unhealthy follicles after ivF cultures (degenerated follicles). Scale bar: 200 μm. Created with BioRender.com.

The degenerated follicles that had lost the 3D microarchitecture, extruded the oocytes, and/or that displayed a darkness aspect of the somatic compartment were discarded (Figure 2).

On the contrary, the following analyses collected the healthy follicles classified as having either “no antrum” or “early antral” (EA) structures based on the follicular 3D microarchitecture with a translucent oocyte and follicular cells with the absence or presence of a preliminary follicular cavity (antrum).

The EA follicles were exclusively considered to isolate the cumulus-oocyte complexes (COCs) that were to be enrolled into the IVM protocols while the gene expression analyses were carried out on the in vitro follicle walls collected from the no-antrum and antrum-healthy structures, as well as from the in vivo PA and EA follicles; the same replicates of the follicles grown in vitro. In more detail, the total RNA was extracted from the single-follicle walls using a Single-Cell RNA Purification Kit (Norgen Biotek Corp. Cat. No. 51800, Thorold, ON, Canada). Then, 0.5 μg of the total RNA was retrotranscribed using oligodT primers (Cat. No. BIO-38029 Bioline, London, UK) and Tetro Reverse Transcriptase (Cat. No. BIO-65050 Bioline, London, UK). qPCRs were carried out in triplicate using the SensiFAST SYBR Lo-ROX kit (Cat. No. BIO-94005 Bioline, London, UK) on a 7500 Fast Real-Time PCR System (Cat. No. 4351107 Life Technologies, Carlsbad, CA, USA), according to the manufacturer’s instructions. The following PCR conditions were used for all of the experiments: 95 °C for 10 min, followed by 40 cycles at 95 °C for 10 s and 60 °C for 30 s. Relative quantification was performed by using the ∆∆Ct method. *GAPDH* (Glyceraldehyde 3-phosphate dehydrogenase) and *YWHAZ* (Tyrosine 3-Monooxygenase/Tryptophan 5-Monooxygenase Activation Protein Zeta) were selected amongst the housekeeping genes for gene quantification. The expression profiles were similar with both reference genes. The primer sequences are reported in Supplementary Table S1.

#### 2.5.2. Meiotic Competence Acquisition of ivF Grown Oocytes

At the end of culture, the COCs were mechanically isolated from the healthy EA follicles with the aid of 32G sterile needles under the stereomicroscope.

Exclusively healthy COCs, classified on the bases of the continuous and compact layers of cumulus cells, and the absence of any signs of ooplasmic degeneration, were used for IVM.

Before performing IVM, the diameters of the oocytes were recorded (time-lapse imaging software NIS-Elements (Eclipse Ti Series, Nikon, Japan).

The COCs isolated from the different protocols (A. 3D-oil, B1. Two-Step PCL-Randomic, B2. Two-Step PCL-Patterned electrospun scaffolds, and C. One-Step PCL-Patterned vs.) were matured into a 4-well plate (Nunc, Roskilde, Denmark) and their meiotic competence was compared with those expressed by COCs derived from the in vivo PA, EA, and antral (A) follicles [87].

In more detail, the COCs were matured for 24 h on a monolayer of ovarian surface epithelium (OSE) cells in the maturation culture medium alphaMEM (Cat. No. BE02-002F Lonza), 20% fetal bovine serum (FBS: Cat. No. 11573397 Gibco), 1% glutamine (Cat. No. BE17-605E/U1 Lonza), antibiotics such as 75 mg/L of penicillin-G and 50 mg/L of streptomycin sulfate (Cat. No. DE17-602E Lonza), hCG, and eCG 10 IU/Petri) [86] prior to the treatment for the nuclear-stage assessment.

To this aim, the COCs were denuded from the cumulus cells under a stereomicroscope, the isolated oocytes were permeabilized/fixed in a solution of acetic acid and ethanol (1:3) for at least 12 h and finally stained with 1% Lacmoid (Cat. No. 274720 Sigma) in distilled water [14]. Then, the oocytes were mounted on the object slide and analyzed under a Phase Contrast Microscope (AxioVert, Carl Zeiss, Jena, Germany) for the detection of the nuclear stage (germinal vesicle-GV, metaphase one-MI, and metaphase two-MII).

### 2.6. Statistical Analysis

Three independent biological replicates were performed. The data are presented as the percentage or mean ± SD. GraphPad Prism 9 (GraphPad Software) was used for the statistical analyses, and values with *p <* 0.05 were considered statistically different.

Differences in antrum formation, the percentage of degenerated follicles, and the achievement of oocyte meiotic competence in vitro between the different treatments were evaluated by ordinary one-way ANOVA followed by the Tukey–Kramer test for comparison of multiple groups. All of the other data were analyzed by an unpaired *t*-test.

## 3. Results

### 3.1. Patterned PCL Topology Enabling Long Term In Vitro Follicle PA Development in Ovine Model

#### 3.1.1. Two-Step PCL Patterned In Vitro Follicles Culture Protocols Was Able to Support PA Follicle Growth and Antrum Formation

**Figure 3:**
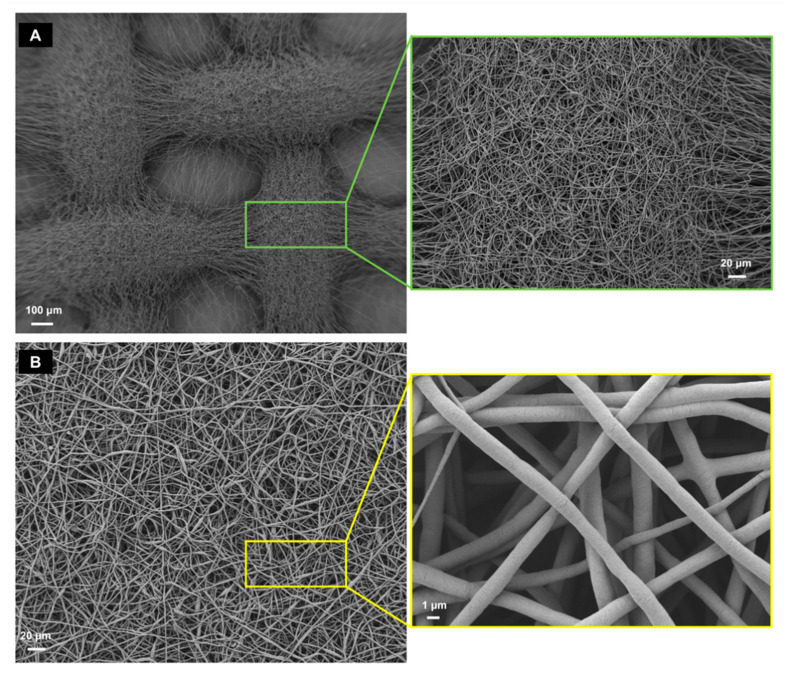
SEM analysis of the PCL-Patterned (**A**) and PCL-Randomic (**B**) scaffolds. PCL-Patterned scaffold is reported in A and its zoomed view is shown in a green frame. The scale bars are 100 μm (magnification 100×) and 20 μm (magnification 500×), respectively. The PCL-Randomic is reported in the bottom (**B**) (magnification 500×) and a higher magnification (10 k× image is shown in a yellow frame. For those images the scale bars are 10 μm and 1 μm, respectively.

**Figure 4:**
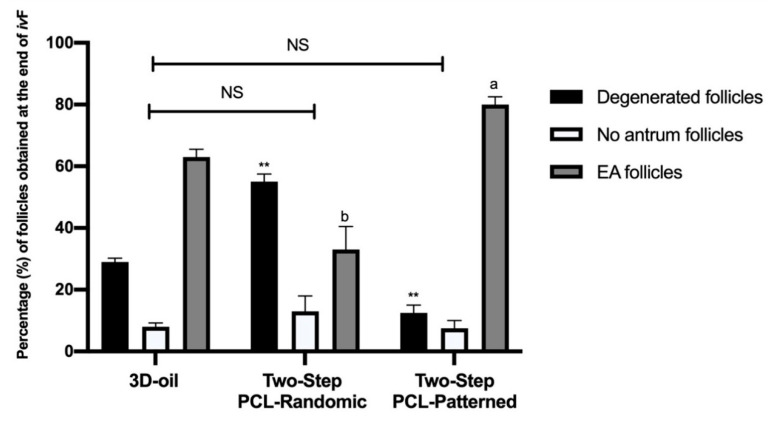
Percentage (%) of degenerated, no antrum and EA follicles per groups at the end of ivF culture. A total of 120, 125, and 122 follicles were cultured in three independent experiments during which 3D-oil, Two-Step PCL-Randomic and Two-Step PCL-Patterned electrospun scaffolds were simultaneal compared (3D-oil: 40, 40 and 40 PA follicles per experiments; Two-Step PCL-Randomic: 41, 42 and 42 follicles PA follicles per experiments; Two-Step PCL-Patterned: 41, 40, and 41 PA follicles per experiments). For each iv*F* group (3D-oil, PCL-Randomic and PCL-Patterned electrospun scaffolds) 75, 39 and 96 EA follicles as well as 24, 44, and 10 degenerated follicles were collected at the end of in vitro culture, respectively. ^a,b^: statistically significant vs. EA follicles of the 3D-oil group (*p* < 0.05 and *p* < 0.01, respectively); **: statistically significant vs. degenerated follicles of the 3D-oil group (*p* < 0.01). NS: not statistically significant.

The obtained electrospun scaffolds were characterized by a comparable average fiber diameter of around 1 μm, and two different topologies, as reported in Figure 3. In fact, Figure 4 shows that the PCL-Patterned sample is characterized by a pattern with macropores of an average size of 300 μm.

Each macropore is crossed by a thin layer composed of few fibers, which allows an improved follicle adhesion [33]. In contrast, the PCL-Randomic samples are composed of randomly oriented networking fibers.

Those two PCL electrospun scaffolds (PCL-Randomic and Patterned) were used for ivF. In more detail, the scaffold-based culture protocol was carried out preliminarily, adopting a Two-Step approach by starting the PA follicle incubation on the 3D-oil-validated system and then individually transferring the growing follicles onto the trans-well culture system filled with PCL-Randomic or PCL-Patterned scaffolds before antrum cavity formation.

The in vitro performances of the PA follicles cultured on the two-step vs. the 3D-oil-validated protocols were compared on day 14. First of all, the incidence of degenerated, no antrum, and EA follicles were recorded (Figure 4).

A very high percentage of degenerated follicles was recorded in the PA follicles grown on PCL-Randomic scaffolds (55%), whereas they were 29% and 12.5% under 3D-oil and PCL-Patterned protocols, respectively (vs. PCL-Randomic *p =* 0.0002 and *p <* 0.0001, respectively).

Of note, the majority of healthy follicles (87.5%) obtained in the Two-Step PCL-Patterned ivF procedure (71%: *p =* 0.0003 and 45%: *p <* 0.0001, for 3D-oil and PCL-Randomic, respectively) were EA follicles (80%). On the contrary, only 63% and 33% of them were able to differentiate antrum under the 3D-oil (*p =* 0.0154) and Two-step PCL-Randomic (33%: *p <* 0.0001) ivF protocols, respectively.

**Figure 5:**
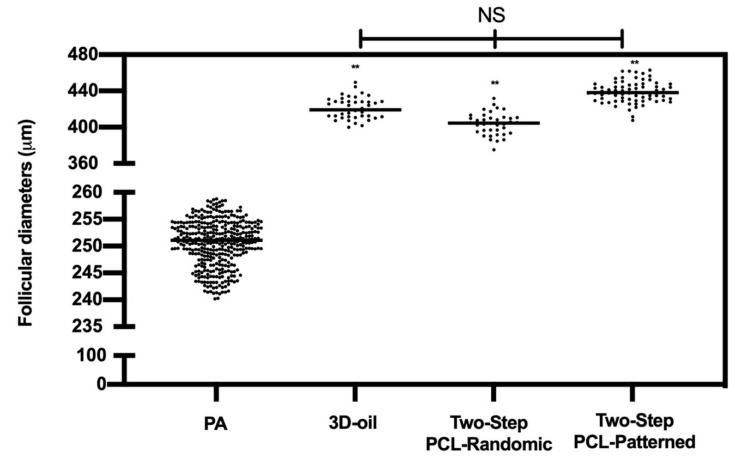
EA follicular mean final diameters: overview for each experimental group. A total of 120, 125, and 122 follicles were cultured in three independent experiments during which 3D-oil, Two-Step PCL-Randomic, and Two-Step PCL-Patterned electrospun scaffolds were simultaneal compared (3D-oil: 40, 40, and 40 PA follicles per experiments; Two-Step PCL-Randomic: 41, 42, and 42 follicles PA follicles per experiments; Two-Step PCL-Patterned: 41, 40, and 41 PA follicles per experiments). A total of 367 follicles (showed in the graph as black dots) were measured to determine the starting mean diameters (PA). Then, 75, 39, and 96 EA follicles, respectively for 3D-oil, Two-Step PCL-Randomic, and Two-Step PCL-Patterned were measured at the end of ivF culture to determine the final mean diameters. **: statistically significant vs. PA group (*p* < 0.01). NS: not statistically significant.

In addition, the follicle growth parameters of the PA follicles used for the ivF protocols (mean diameter of 250.5 ± 4) were analyzed at the end of the ivF culture (Figure 5 and Table 1).

The final diameter of the healthy follicles at the end of the culture was approximately 400 µm, independent of the protocols adopted (3D oil vs. PCL-Randomic and PCL-Patterned adopted (for both *p* > 0.05; see Table 1 and Figure 5) and of the ability of the follicles to develop an antrum (Table 1).

**Figure 6:**
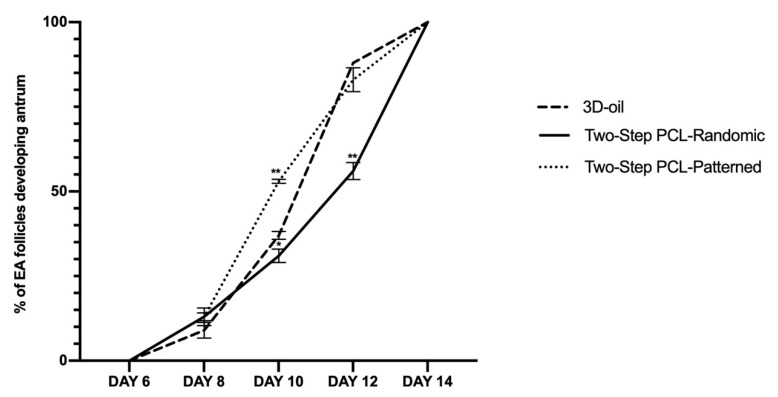
Development trend of the follicular antrum over time according to follicular treatment groups. A total of 120, 125, and 122 follicles were cultured in three independent experiments during which 3D-oil, Two-Step PCL-Randomic, and Two-Step PCL-Patterned electrospun scaffolds were simultaneal compared (3D-oil: 40, 40 and 40 PA follicles per experiments; Two-Step PCL-Randomic: 41, 42, and 42 follicles PA follicles per experiments; Two-Step PCL-Patterned: 41, 40, and 41 PA follicles per experiments). Specifically, only follicles that differentiated the follicular antrum during ivF cultures, were tested (75, 39, and 96 EA follicles, respectively, for 3D-oil, Two-Step PCL-Randomic, and Two-Step PCL-Patterned electrospun scaffolds). *, **: statistically significant vs. 3D-oil group (*p* < 0.05 and *p* < 0.01, respectively). Data in which subscripts are not indicated, were considered not statistically significant.

Moreover, differences in the kinetics of the antrum formation were recorded among the different culture protocols (Figure 6).

Any sign of antrum formation was observed until the day of follicle transfer onto the scaffold (day 6). The first evidence of antrum occurred in a very low percentage of the follicles on day 8 (<10%) and then dramatically increased on day 10 (see Figure 6).

In particular, the antrum formation was accelerated in the Two-Step PCL-Patterned group (53% vs. 31% of PCL-Randomic and vs. 37%; 3D oil at day 10, for both *p* < 0.0001). A major delay was recorded in the PCL-Randomic group (55% of antrum in PCL-Randomic vs. 83% 3D-oil and vs. 88% PCL-Patterned at Day 12: for both *p* < 0.0001).

#### 3.1.2. Follicle Gene Expression Profile Was Positively Regulated by Two-Step PCL-Patterned In Vitro Follicles Culture-Based Protocols

**Figure 7:**
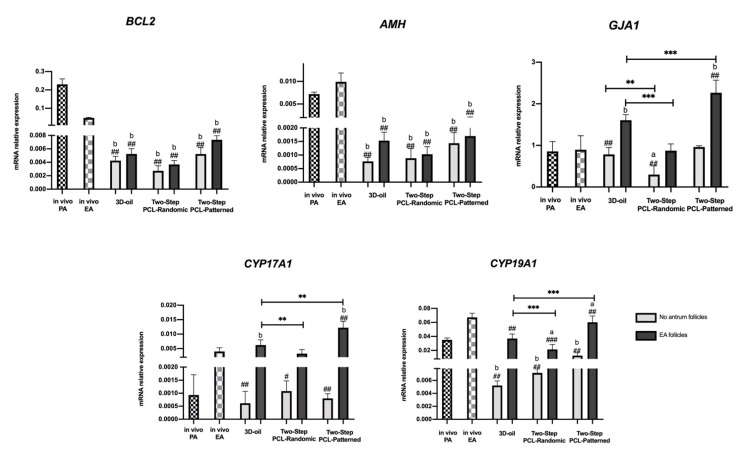
Representative expression of steroidogenic and somatic-specific genes. Twelve follicular walls per group were processed for gene expression analysis. Data were expressed as mean ± SD. **, ***: statistically significant vs. 3D-oil group (*p* < 0.05, *p* < 0.01 and *p* < 0.0001, respectively). #, ##, ###: statistically significant vs. in vivo EA follicles (*p* < 0.05, *p* < 0.01 and *p* < 0.0001, respectively). ^a,b^: statistically significant vs. in vivo PA follicles (*p* < 0.05 and *p* < 0.01, respectively). Data in which subscripts are not indicated were considered not statistically significant.

The steroidogenic transcript program activation (*CYP17A1* and *CYP19A1*) was assessed since they would not be able to accurately detect the quantity of hormones released from the growing follicles in the cultural media. Follicle development was also studied by analyzing three other representative genes regulating in vitro follicle growth (*BCL2*, *AMH,* and *GJA1*: Figure 7).

The *BCL2* and *AMH*, that are both physiologically modulated during the transition from PA to EA (4.9 reduced and 1.28-fold increased, respectively), displayed very low levels of expression at the end of ivF, independently of the protocols used (for *BCL2*: 54, 85 and 46-fold decreased, respectively, for 3D-oil, Two-PCL Randomic, and Two-PCL Patterned no antrum categories; 46, 63.8, and 31.3-fold decreased respectively for 3D-oil, Two-PCL Randomic and Two-PCL Patterned EA categories; for *AMH*: 10, 8.75 and 5-fold decreased respectively for 3D-oil, Two-PCL Randomic and Two-PCL Patterned no antrum categories; 4.6, 5.4 and 4-fold decreased, respectively, for 3D-oil, Two-PCL Randomic, and Two-PCL Patterned EA categories).

The in vitro grown follicles always displayed an upregulation of *GJA1*, which was significantly higher in the EA follicles cultured in the Two-Step PCL-Patterned protocol (2.75-fold change increase and 1.4-fold change increase over PCL-Randomic and 3D-oil, respectively: *p* < 0.0001). Moreover, *GJA1* expression was strictly related to the process of antrum cavity formation. Indeed, in all of the tested culture conditions, a significant increase in gene expression was obtained in the EA follicles: in particular, the PCL-Patterned had a 2.4-fold change increase, PCL-Randomic 2.9-fold, and the 3D-oil had a 2-fold change increase over the no-antrum follicle categories (*p* < 0.0001).

Interestingly, the expression of steroidogenic-related genes (*CYP17A1* and *CYP19A1*), which physiologically increased during the transition from the PA to the EA stage (2.8-fold change increase for *CYP17A1* and 1.7-fold for *CYP19A1*) followed a similar trend and amplitude, particularly in the Two-Step PCL-Patterned scaffold (Figure 7). There were no significant differences between in vivo and in vitro EA for *CYP19A1* expression, as well as a slight increase in *CYP17A1* was detected in only the Two-Step PCL-Patterned protocol. Furthermore, the process of antrum cavity formation was strictly related to the activation of steroidogenic enzymes as well as of the other follicular gene expressions, independent of the culture conditions.

#### 3.1.3. PCL-Patterned Scaffolds Support a Synergic Follicle and Oocytes Development

**Figure 8:**
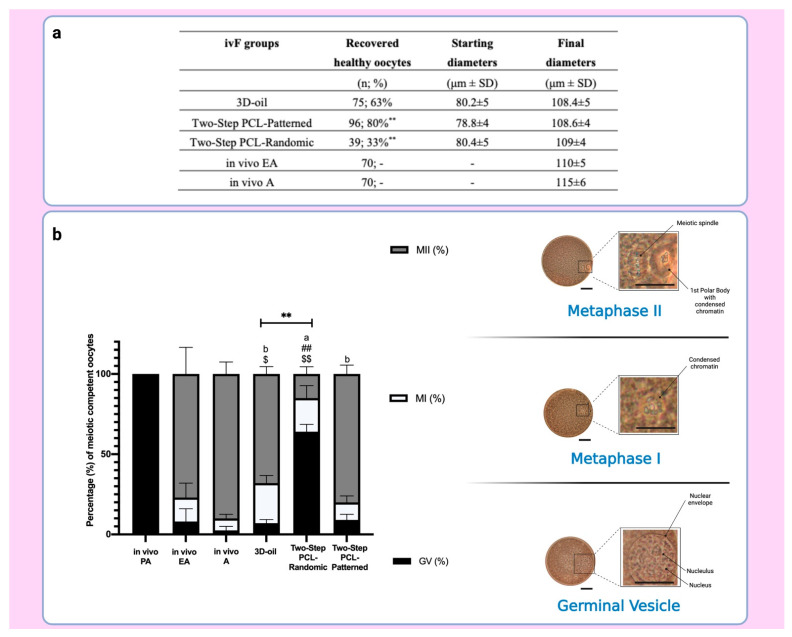
(**a**) Table summarizing the number of recovered healthy oocytes and their mean diameters at the end of ivF culture. **: statistically significant vs. 3D-oil group. (**b**) On the left: Percentage of the oocyte meiotic competence acquired in vitro. Different nuclear stages were characterized. ^a,b^: statistically significant vs. recovered healthy oocytes from in vivo PA (*p* < 0.05 and *p* < 0.01, respectively); ##: statistically significant vs. recovered healthy oocytes from in vivo EA (*p* < 0.01); $, $$: statistically significant vs. recovered healthy oocytes from in vivo A (*p* < 0.05 and *p* < 0.01, respectively); **: statistically significant vs. recovered healthy oocytes from 3D-oil group (*p* < 0.01). Data in which subscripts are not indicated, were considered not statistically significant. On the right: Images of oocyte nuclear stages. GV: Germinal Vesicle Break Down; MI: Metaphase 1; MII: Metaphase II (Lacmoid solution staining, 40× magnification: scale bar: 30 μm; insert of the oocytes details; scale bar 30 μm). Created with BioRender.com.

A higher percentage of healthy oocytes were collected from the EA follicles developed under the Two-Step PCL-Patterned-based ivF protocol (80% vs. 33% vs. 63% PCL-Patterned, PCL-Randomic and 3D-oil-derived oocytes, respectively: Figure 8). There were no significant differences between the final oocyte diameters between culture approaches (see Figure 8a).

The assessment of the nuclear stage after IVM showed a significantly higher incidence of MII oocytes retrieved from the follicles cultured on the PCL-Patterned electrospun scaffolds (80%) than on the PCL-Randomic (15%, *p* < 0.0001) and 3D-oil systems (68%, *p* = 0.04; see Figure 8b), respectively. Of note, the overall meiotic competence displayed from the oocytes isolated from follicles grown on the PCL-Patterned scaffold was comparable to that of the oocytes enclosed in the in vivo EA ones (80% vs. 77%, respectively) (Figure 8a). Of note, the lowest ability to resume meiosis (36% vs. 91% and 93% of PCL-Patterned and 3D-oil, respectively; *p* < 0.0001) was displayed by the PCL-Randomic-related oocytes.

### 3.2. Two and One-Step PCL-Patterned-Based Protocols Showed the Same Ability to Support the Synergic In Vitro Growth of Follicle and Oocyte Compartments

#### Similar Morphological Performances of In Vitro Grown PA Follicles Cultured under Two- and One-Step PCL-Patterned In Vitro Follicles Culture Protocols

To verify if follicle development can be supported in vitro even by adopting a 14-day trans-well protocol based on the use of a PCL-Patterned electrospun scaffold (One-Step PCL-Patterned system), further experiments were carried out in order to compare the PA follicle and oocyte performances grown under the Two vs. One -Step PCL-Patterned system.

**Figure 9:**
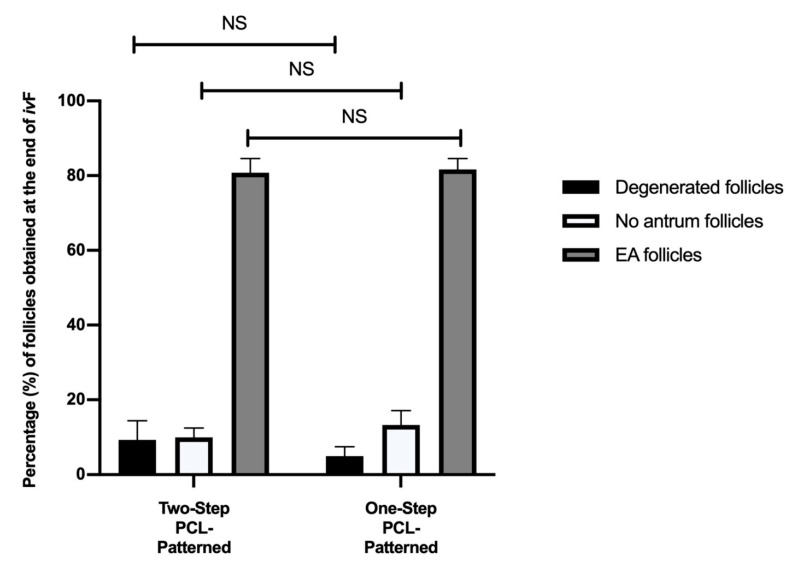
Percentage (%) of degenerated, no antrum and EA follicles per groups at the end of ivF culture. A total of 120 and 120 follicles were cultured in three independent experiments during which Two-Step PCL-Patterned and One-step PCL-Patterned electrospun scaffolds were simultaneal compared (Two-Step PCL-Patterned: 40, 40, and 40 PA follicles per experiment; One-Step PCL-Patterned: 40, 40, and 40 PA follicles per experiment). NS: not statistically significant.

As reported in Figure 9, the assessment of the morphological parameters showed that both the Two- and One-Step PCL-Patterned systems were able to support in vitro follicle development without displaying any differences in terms of degenerated, healthy, and antrum follicles’ percentages.

**Figure 10:**
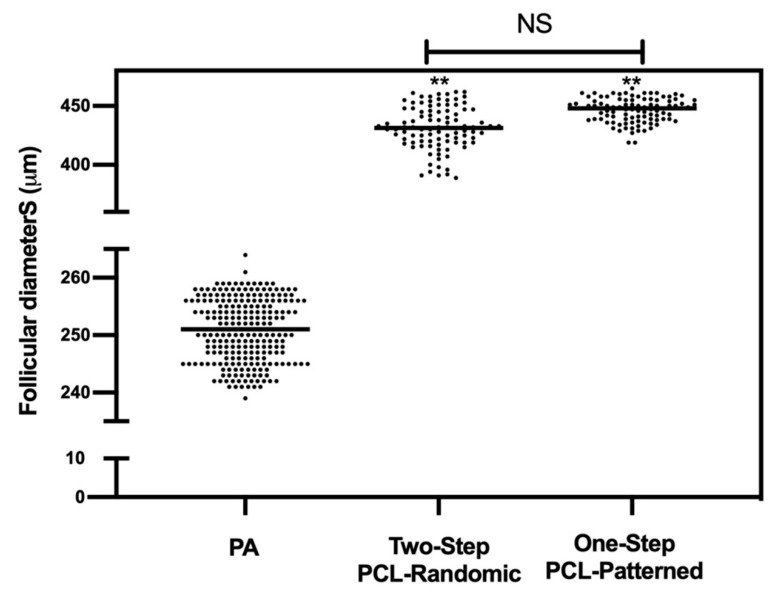
EA follicular mean final diameters: overview for each experimental group. A total of 120 and 120 follicles were cultured in three independent experiments during which Two-Step PCL-Patterned and One-step PCL-Patterned electrospun scaffolds were simultaneal compared (Two-Step PCL-Patterned: 40, 40, and 40 PA follicles per experiment; One-Step PCL-Patterned: 40, 40, and 40 PA follicles per experiment). **: statistically significant vs. PA follicles group (*p* < 0.01). NS: not statistically significant.

**Figure 11:**
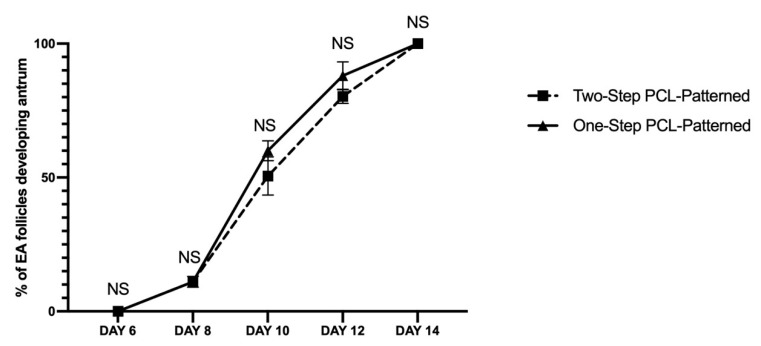
Development trend of the follicular antrum over time according to follicular treatment groups. A total of 120 and 120 follicles were cultured in three independent experiments during which Two-Step PCL-Patterned and One-step PCL-Patterned electrospun scaffolds were simultaneal compared (Two-Step PCL-Patterned: 40, 40, and 40 PA follicles per experiment; One-Step PCL-Patterned: 40, 40, and 40 PA follicles per experiment). Specifically, only follicles that differentiated the follicular antrum during ivF cultures, were tested (97 and 98 EA follicles, respectively, for Two-Step PCL-Patterned and One-Step PCL-Patterned). NS: not statistically significant.

Analogously, similar performances were observed at the morphological (diameters; Figure 10) and functional (antrum formation) levels in the follicles collected at the end of both of the ivF protocols (Two vs. One-Step). In more detail, the majority of follicles (90.8% and 95% for Two-Step and One-Step PCL-Patterned, respectively) were healthy, and the EA displayed a mean final diameter of approximately 450 μm independently of the protocol (Two vs. One step: *p* > 0.005). No significant differences were also observed in the kinetic of follicular antrum development over time (Figure 11).

A total of 240 follicles were measured to determine the starting mean diameters (PA). Overall, 97 and 98 EA follicles, respectively, for the Two-Step PCL-Patterned and One-Step PCL-Patterned were measured at the end of ivF culture to determine the final mean diameters.

### 3.3. Steroidogenic and Somatic Specific Gene Expression on In Vitro Follicles Culture: Two-Step vs. One-Step PCL-Patterned System

**Figure 12:**
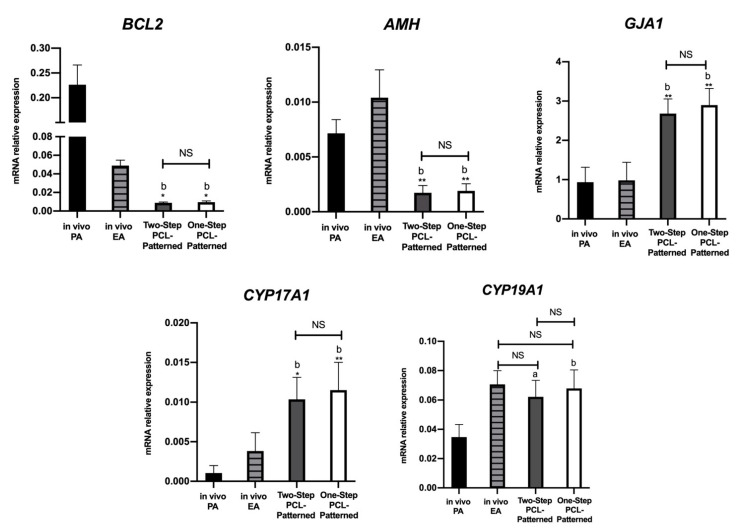
Representative expression of steroidogenic and somatic-specific genes. Twelve follicular walls per group were processed for gene expression analysis. Data were expressed as mean ± SD. ^a,b^: statistically significant vs. in vivo PA follicles group (*p* < 0.05 and *p* < 0.01, respectively). *, **: statistically significant vs. in vivo EA follicles group (*p* < 0.05 and *p* < 0.01, respectively). NS: not statistically significant.

The analysis of the gene expression profile of the somatic compartments derived from both of the ivF scaffold-based systems revealed a slight increase in the gene transcripts’ levels of follicles from the One-Step PCL-Patterned protocol that, however, did not reach any significance. The PCR data confirmed that the *BCL2*, *AMH*, and *GJA1* gene expression profiles of both the Two- and One-Step-derived follicles diverged from the in vivo ones (Figure 12). The in vivo EA category has been characterized by down-regulation (4.7-fold change) and upregulation (1.42-fold change increase in *BCL2* and *AMH*, respectively).

On the contrary, the *GJA1* transcripts that were not physiologically affected by the transition from PA to EA follicles were always increased in follicle compartments derived from both the Two- and One-Step protocols. Furthermore, *CYP17A1* expression was sustained in an in vitro context (2.7-fold change increase and 3-fold change increase for Two-Step PCL-Patterned and One-Step PCL-Patterned compared to in vivo EA), whereas any significant modulation between in vitro and in vivo was detectable for *CYP19A1*. Focusing on the in vivo system, the *CYP17A1* and *CYP19A1* genes were significantly upregulated in the EA follicles, with a 3.8-fold change increase in *CYP17A1* and a 2.3-fold change increase in *CYP19A1*, respectively.

### 3.4. Both Two and One-Step-Derived Oocytes Achieved on Fibrous Patterned Scaffolds a Complete Meiotic Competence

**Figure 13:**
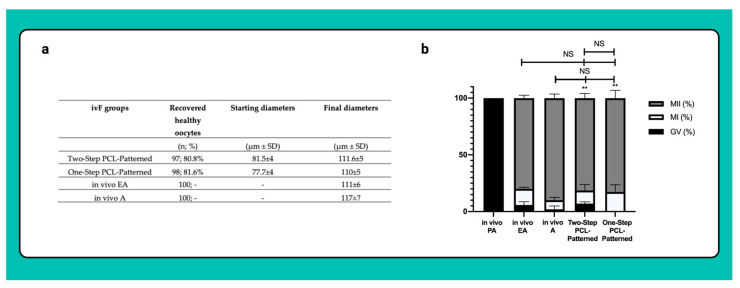
(**a**) Table summarizing the number of recovered healthy oocytes and their mean diameters at the end of ivF culture. A total of 120 and 120 follicles were cultured in three independent experiments during which Two-Step PCL-Patterned and One-step PCL-Patterned electrospun scaffolds were simultaneal compared (Two-Step PCL-Patterned: 40, 40, and 40 PA follicles per experiment; One-Step PCL-Patterned: 40, 40, and 40 PA follicles per experiment). For each ivF group (Two-Step PCL-Patterned and One-Step PCL-Patterned), 97 and 98 healthy oocytes were recovered and analyzed at the end of the in vitro culture to test the oocyte performance. Only the oocytes retrieved from follicles that differentiated the follicular antrum, were tested; data are expressed as mean ± SD. (**b**) Percentage of the oocyte meiotic competence acquired in vitro. Different nuclear stages were characterized. **: statistically significant vs. in vivo PA follicles group (*p* < 0.05). NS: not statistically significant. Data in which subscripts are not indicated, were considered not significant. GV: Germinal Vesicle; MI: Metaphase I; MII: Metaphase II.

The vast majority of oocytes isolated from the EA follicles were healthy and reached a final diameter of approximately 110 μm, independently of the One or Two-Step patterned protocol (Figure 13a).

As for the oocytes isolated from the in vivo EA and in vivo A follicles, more than 80% of the gametes derived from the Two- and One-Step ivF scaffold-based protocols reached the MII stage at the end of IVM (81.4% vs. 83%; respectively) while the remaining oocytes resume meiosis without completion (11.6% and 17.3% MI, respectively). Of note, exclusively, the oocytes retrieved from follicles cultured on One-Step PCL-Patterned were all able to resume meiosis, whereas 5.8% and 7% of those isolated from follicles derived from the Two-Step PCL-Patterned or from the in vivo EA ones remained in the GV stage (*p* = 0.0102 and *p* = 0.0292 vs. One-Step, respectively; Figure 13b).

## 4. Discussion

Tissue engineering provides a means to apply the principles of biology and biomedical engineering in order to develop functional supports that are able to replace compromised or damaged tissue [88,89]. In the context of reproductive tissue engineering (REPROTEN), biomaterial scaffolds appear to be promising choices for repairing reproductive tissues and avoiding the potential complications of donor-site morbidity related to the transplant process [34]. Of note, the REPROTEN branch has received great interest from the scientific community since tissue engineering scaffold-based approaches for the regeneration of reproductive organs and tissues [28,87] are potentially suitable for pediatric oncological patients and in women suffering from premature ovarian failure, and as they can be exploited to amplify the reproductive performances of domestic animals and preserve endangered species that, to date, remain a challenging target of ART. In this way, the use of a so-called “artificial ovary” could prove to be a promising way to overcome those limits dictated by in vitro folliculogenesis protocols, even though they may conceivably offer the opportunity to reproduce in vitro the growth of a large pool of immature gametes enclosed in the follicles during the early stages of development by obtaining fully-grown meiotically competent oocytes potentially able to generate live births. Currently, greater efforts are needed to fully recapitulate the ovarian folliculogenesis process in vitro. In fact, the ivF culture approach tries to translate what physiologically occurs in vivo in an in vitro context, and this would require more effort in terms of ivF protocol efficiency. In addition, the role of two and three-dimensional follicle culture in vitro approaches has been previously investigated [22], the production of live offspring from primordial follicles cultured in vitro has been successfully achieved only in a murine model [90], whereas in medium-large mammals, the in vitro production of a fertilizable oocyte from a primordial follicle requires a long-term culture, which may affect oocyte quality and, consequently, embryo production [83,91]. The main limitation of ivF efficiency is given the struggle to recapitulate the in vitro process of ovarian folliculogenesis, which in species other than murine one, require high-quality standards and the use of culture approaches that still need to be optimized and investigated. Nonetheless, the advantage of reproducing an artificial ovary using engineered biomimetic scaffolds, which present biocompatible polymers that act as main actors of follicular and oocyte well-being in vitro, would allow the preservation of the native follicular morphological structure, allowing the follicles to grow in an environment as similar to the physiological one. Within this framework, the present work aimed to highlight the pivotal role of biomaterial properties and the related follicle–biomaterial interactions in an in vitro developmental context, comparing the results to a previously validated approach [14,86] and focusing attention on the electrospinning technique for fiber fabrications, which previously has been shown to be an appropriate technique to obtain a fibrillary morphology similar to the native ovarian cortex in a porcine model [35,36,79]. Moreover, the relevance of the fiber structure in the human ovary has been highlighted in recent work by Ouni et al. [92]. Likewise, poly (epsilon-caprolactone) (PCL)-based electrospun randomic and patterned scaffolds were investigated as support for ovine preantral follicles in vitro development, evaluating the follicular and oocyte performance in terms of follicle and oocyte growth, as well as the follicle capability to develop antrum cavity, gene expression profile, and the achievement of oocyte meiotic competence. Based on our results, PCL-Patterned electrospun fibrous scaffolds have shown a remarkable ability to guide follicular morphology and affect oocyte and follicular performance in vitro when compared to PCL-Randomic electrospun fibrous scaffolds. In fact, the highest percentage of healthy follicles, which have not lost their spherical structure at the end of ivF culture, was recorded in follicles grown on Two-Step PCL-Patterned, showing a recovery percentage of 87.5% compared to Two-Step PCL-Randomic (45%; *p <* 0.0001) and 3D-oil protocol (71%; *p =* 0.0003). Similarly, the developmental rate of the antrum cavity appears to be significantly marked in follicles that have grown on the Two-Step PCL-Patterned scaffolds (80% vs. 33% and 63%, respectively, for Two-Step PCL-Randomic *p <* 0.0001 and 3D-oil *p =* 0.0154), highlighting how, among other culture approaches, the follicular antrum developmental trend over time is accelerated in follicles cultured on patterned fibers on day 10 of ivF culture (53% vs. 31% of PCL-Randomic and vs. 37% of 3D oil; *p <* 0.0001) denoting, on the other hand, a considerable delay in the antral cavity formation in follicles grown on the Two-Step PCL-Randomic scaffolds (55% of antrum in PCL-Randomic vs. 83% 3D-oil and vs. 88% PCL-Patterned at Day 12: for both *p <* 0.0001). Interestingly, the acceleration of follicular antrum development in follicles grown in the PCL-Patterned approach is directly proportional to the ability of the follicles themselves to positively regulate the expression profile of some representative genes in the intercellular cooperation between cumulus cells and oocyte and in the regulation of follicular and steroidogenic activity in vitro. Specifically, the upregulation of *GJA1*, which plays a pivotal role in the formation of communication channels between the somatic and germinal compartments during follicular development was significantly higher I then EA follicles developed under the Two-Step PCL-Patterned protocol compared to the Two-Step PCL-Randomic and 3D-oil approach (*p <* 0.0001). Indeed, the culture conditions induced a higher *GJA1* expression compared with the in vivo conditions, likely ensuring improved coordination and synchronization of the processes involved in granulosa cell proliferation and differentiation [85,93]. Therefore, the greater expression of *GJA1* could indirectly suggest that in vitro follicle growth on PCL-Patterned scaffolds guarantees an efficient coupling between the somatic and germinal compartments, justified by the results obtained regarding the performance of meiotic competence acquisition and the activation of steroidogenic enzymes. Considering this last aspect, the cooperation between the germinal and somatic compartments assumes an intervention at trophic and growth levels given both by theca and granulosa cells since the interaction between them is essential for the production of steroid hormones [94,95,96]. In light of this, the determination of the steroidogenic potential of follicles cultured in vitro with the tested different culture approaches was performed by assessing the transcriptional activation program of the *CYP17A1* and *CYP19A1* genes, key step points in steroid biosynthesis. Considering the physiological aspect, granulosa cells act cooperatively with theca cells in estrogen synthesis, as well as the synthesis of steroid hormones and the maturation of oocytes promote the maintenance of female reproductive performances [97,98]. Based on the obtained findings, the expression of steroidogenic enzymes physiologically increases during the transition from PA to EA follicles (see Figure 8), denoting a similar trend in follicles cultured on PCL-Patterned scaffolds that developed an antrum cavity compared to other approaches. This result confirms a pivotal point in suggesting that the follicular antrum formation supports the modulation of theca specific gene *CYP17A1* expression since it is denoted by an upregulation of this gene in the EA follicle category, specifically in those follicles cultured on patterned electrospun fibers. Moreover, regarding the *CYP19A1* gene that provides instruction for making the aromatase enzymes, its upregulation could be interpreted as a consequence of the boosted production of precursors for estrogen synthesis, together with synchronous crosstalk between oocyte, granulosa, and the theca cells, which proves to be a relevant aspect in consideration of follicular and oocyte well-being. Taking into consideration the metabolic coupling between somatic and germinal compartments, the ability to resume meiosis and the degree of meiotic competence acquisition of the oocytes recovered from EA follicles grown in the different culture approaches was evaluated in order to support the thesis that a coherent expression of the genes responsible for follicular development and steroidogenetic activity in vitro can be translated into a potential capability of the oocytes faced with an in vitro fertilization process. Our findings showed that a significantly higher percentage of MII oocytes was recorded from follicles in vitro grown on the Two-Step PCL-Patterned electrospun scaffolds (80%) compared to those cultured on the Two-step PCL-Randomic (15%; *p <* 0.0001) and in 3D-oil approach (68%; *p =* 0.04; see Figure 8a). Moreover, the use of PCL-Patterned electrospun fibers as functional support for in vitro follicle development displayed a performance of meiotic competence acquisition similar to that recorded in oocytes collected from in vivo EA follicles (80% vs. 77%, respectively). Of note, it is relevant to highlight how the ability to resume meiosis is a condition that occurs in a reduced percentage in follicles grown on Two-Step PCL-Random fibers (36%) as compared to the Two-Step PCL-Patterned and 3D-oil system (91% and 93%, respectively), assuming that the random distribution of PCL fibers would not support the metabolic and functional dialogue between theca/granulosa cells and oocyte, in order to ensure a follicular development as similar to that which would be reached in physiological conditions, furthermore, supported by the aforementioned levels of gene expression.

In light of the gathered data, to establish if in vitro PA follicle growth can be sustained even by adopting a 14-day trans-well approach using the PCL-Patterned electrospun scaffold (One-Step PCL-Patterned system), further experiments were carried out to compare the follicular and oocyte performances under the Two- vs. One-Step PCL-Patterned protocols. Our findings suggested that the Two- and One-Step PCL-Patterned approaches were able to guarantee the synergic follicular-oocyte development in vitro, showing similar performances considering morphological parameters such as follicular quality and growth diameters, number of healthy follicles recovered after ivF culture protocols, and the kinetics of antrum cavity formation. Based on steroidogenic and somatic specific gene expression, the analysis showed a slight increase in gene transcript levels in the EA follicles derived from the One-Step PCL-Patterned approach; any significant differences were recorded between the two different culture protocols. Moreover, also for this comparison, the data confirm a divergence with respect to the in vivo system for the three representative somatic genes regulating in vitro follicle growth: *BCL2*, *AMH*, and *GJA1*. Based on our results, both the Two-Step and One-Step PCL-Patterned protocols were characterized by a down-regulation and upregulation of *BCL2* and *AMH*, respectively, in the EA follicles recovered after the ivF cultures. In regards to the expression of the *GJA1* gene, a marked increase in the follicular compartments derived from both culture approaches is confirmed. These findings can suggest that the expression of *GJA1* facilitates intercellular communication among growing oocytes and surrounding follicular cells regardless of the days of follicular culture in vitro for the Two-Step and One-Step approaches, respectively, thereby connecting the developing gamete to an essential network of supporting granulosa and theca cells. To confirm this, it is possible to note how the expression of *CYP17A1* was supported in vitro for both culture approaches compared to the in vivo system, suggesting that its upregulation resulted in an early and fast differentiation status of follicle development sustained by the in vitro culture protocols. This advanced follicular cell differentiation reflects the early ability of the oocytes derived from EA follicles cultured on PCL-Patterned electrospun fibers to resume meiosis and to be able to achieve meiotic competence. In fact, both culture approaches show a high degrees of oocyte meiotic competence recovery, assuming that these findings are the result of an active and coordinated dialogue between the different cellular compartments aimed at supporting the oocyte nourishment and the different phases of follicular development in vitro that mediate the external signals during its maturation process. Notably, the three-dimensional structure of the ovarian follicle, which allows the maintenance of synergistic connections between somatic cells and the gamete, is crucial for the successful growth and maturation of the oocyte [99,100]. Accordingly, the 3D cell culture systems represented a better choice to preserve cell-to-cell and cell-to-matrix interaction in maintaining the follicular morphological shape [101,102,103], providing proper steroid production [103,104,105]. In this context, ovarian tissue engineering is presented as a 3D system capable of supporting folliculogenesis in terms of survival and follicular growth [106]. Nevertheless, the biomaterials used for this task require high-quality standards in terms of biocompatibility, biodegradability, and the ability to exchange nutrients and waste products, allowing an appropriate relationship between rigidity and elasticity in maintaining the spherical structure of the follicle and providing an appropriate substrate for its radial growth. For example, natural polymers such as alginate are widely used for scaffold fabrication and for follicle cultures according to their degree of biocompatibility and biodegradability, enhancing the interaction between the cells and biomaterial, as well as improving cell adhesion, migration, and differentiation [29,34,77,78]. However, they usually have poor mechanical properties, which are not comparable to native tissue, and which determine a concrete difficulty in handling the sample during implantation, making it difficult to control the rate of degradation, considering, moreover, that most of them are enzymatically biodegradable [107]. In view of this, synthetic polymers have been mostly used for tissue engineering and regenerative medicine applications, and they are chemically defined materials that allow the synthesis of reproducible scaffolds with tunable properties to guide cellular behavior. Although their use has been little explored in the field of ovarian tissue engineering, Poly(epsilon-caprolactone) (PCL) has proved to be an excellent candidate for this purpose, as previously demonstrated [33,34,77], considering its peculiarity of being an FDA-approved, biodegradable and biocompatible polyesters with many applications in the field of tissue engineering [37,38,39,40,41,42,43,44,45,46,47,48,49,50,51,52,53,54,55,56,57,58,59,60,61,62,63,64,65,66,67,68,69,70,71,72,73,74,75,76]. Therefore, the choice of a suitable biomaterial to be used has a pivotal role in the creation of an artificial ovary in terms of guaranteeing dynamic physiological conditions, mechanical strength, and proteomic compositions. Considering the artificial ovarian morphology, which needs to be as similar as possible to that of the native tissue, in the present work, the electrospinning technique was used for scaffold fabrication with a hierarchical structure composed of submicrometric fibers, with nanoporosities arranged in nets with macropores [33,34,77]. Nevertheless, further investigations are needed on 3D printing and microfluidics technologies to be able to minutely mimic the physiological structure of the ovary, also considering that the ultimate purpose of designing an artificial ovary is precisely to be grafted into the patient. From a literature point of view, all the in vivo studies using an artificial ovarian scaffold were performed in murine models, with reduced transplantation time [108]. The use of a medium-size mammals model, such as ovine, represents a breakout in the field of REPROTEN since it presents numerous advantages in respect to the mouse one, as the length of the follicle maturation period is more comparable to humans with respect to mice model; promising findings were obtained using PCL-Patterned electrospun fibrous scaffolds compared to PCL-Randomic and 3D-oil approach in terms of reduced follicle loss during seeding and follicle survival after a longer time in vitro culture [33,34,77]. Moreover, the PCL-Patterned electrospun scaffold offers the advantage of overcoming the potentially inadequate nutrient supply of follicles cultured on 3D-oil systems, in which the follicle development is limited by the small volume of the culture medium [109]. Consequently, the PCL-Patterned electrospun scaffold would better sustain follicular wellness, guaranteeing high-quality standards in terms of morphology and synergistic crosstalk between the germinal and somatic compartments [14,91,101], which in turn determine the success of the oocyte meiotic potential development within the follicle.

Based on these premises, oocyte IVM can be achieved directly on the scaffold-based culture follicle system once follicular growth is completed in vitro. Moreover, the possibility of transplanting the present system in an in vivo context [33,34,77] would represent a more ambitious future perspective.

Further studies are still ongoing to investigate and improve scaffold–follicle interactions in order to be able to realize the performing follicular qualitative standards obtained in vitro in a totally physiological context, overcoming the still-difficult border between “the concept” and the “concrete approach”, making this next step a great challenge in the world of reproductive engineering.

## 5. Conclusions

The recapitulation of ovarian follicular physiology on a so-called “artificial-ovary” could represent the innovation necessary to overcome the boundary layer between theory and practice, making the use of a biomimetic scaffold not only optimal for tissue regeneration/transplantation purposes but also for reproducing a more physiological environment to support the functional unit of the ovary that would find fertile ground to proceed in its synchronous growth with the gamete enclosed within. The present work, in fact, has confirmed the biomimetic reproductive role of PCL-based electrospun scaffolds by demonstrating for the first time that this biomaterial can also be proposed for ivF purposes. In addition, the ivF added REPROTEN value of patterned PCL scaffold protocol was proven on ovine PA follicles, a high translation animal model that presents several similarities with humans.

The present results seem to confirm that the 3D PCL follicle culture had the advantage of leading to synchronous crosstalk between the somatic and germinal compartments by improving the quality of follicular and oocyte development, causing the border between in vitro and in vivo folliculogenesis to grow closer. Even if further studies are needed to concretize the ART challenge to control every step of the female reproductive cycle, the advancements of bioengineering will allow us now, more than in the past, to build up female reproduction technological strategies in an entirely new way by designing, at the same time, customized solutions that fit with the reference target.

## Figures and Tables

**Table 1:** PCL-electrospun scaffold influence on ivF outcomes: quantitative and qualitative aspects according to follicular experimental groups.

ivF Groups	*N°* PA	PA Diameter	*N°* EA	Mean Diameter of EA Follicles and ∆ Growth (%)	*N°* No Antrum	Mean Diameters of No Antrum Follicles and ∆ Growth (%)
	(*n*)	(μm ± SD)	(*n*)	(%)	(*n*)	(μm ± SD)
3D-oil	120	249 ± 5	75	421.3 ± 12 ** (70%)	21	393 ± 16 ** (67.4%)
Two-Step PCL-Randomic	125	251 ± 4	39	404.3 ± 12 ** (62%) ^b^	42	421.5 ± 16 ** (58%) ^##^
Two-step PCL-Patterned	122	253 ± 6	96	438.5 ± 11 ** (75%) ^a^	16	418 ± 10 ** (67.3%)

EA follicles were analyzed at the end of the in vitro culture for both follicular and oocyte performance. Percentages refer to ∆ growth performances. Follicles diameters are expressed as mean ± SD. **: statistically significant vs. PA diameter groups (*p* < 0.01). ##: statistically significant vs. ∆ growth (%) of no antrum follicles of 3D-oil group (*p* < 0.01). ^a,b^: statistically significant vs. ∆ growth (%) of EA follicles of 3D-oil group (*p* < 0.05 and *p* < 0.01, respectively). Data in which subscripts are not indicated, were considered not statistically significant.

## Data Availability

Not applicable.

## Supplementary Materials

The following supporting information can be downloaded at: https://www.mdpi.com/article/10.3390/cells11121968/s1, Table S1: Sequences of primers used in real-time qPCR.
