# Supplementary material for: When Electrospun Fiber Support Matters: In Vitro Ovine Long-Term Folliculogenesis on Poly (Epsilon Caprolactone) (PCL)-Patterned Fibers
Source: Cells. 2022 Jun 19;11(12):1968. doi: 10.3390/cells11121968 (PMC9222101; doi:10.3390/cells11121968)
Supplement: Supplementary file 1 [file cells-11-01968-s001.zip › cells-1771527-supplementary.pdf]

Table S1. Sequences of primers used in real-time qPCR.

Gene	Forward sequence	Reverse sequence
BCL2	5′-CCTTCTTTGAGTTCGGAG-3′	5′-CTGGCTGTCTCTGAAGG-3′
AMH	5′-GTGGTGCTGCTGCTAAAGATG-3′	5′-CTCCTCATCAGCCTGTCCGA-3′
GJA1	5′-CACTTGAAGCAGATTGAA-3′	5′-AGGATACTGATGATGTAGG-3′
CYP17A1	5′-CTTACCATTGACAAAGGCACAGAC-3′	5′-CAACTCATCTCGCCATCATTAAGC-3′
CYP19A1	5′-TCGTCCTGGTCAACCCTTCTG-3′	5′-CCAGACGAGACCAGAGACCG-3′
GAPDH	5′-TCGGAGTGAACGGATTTGGC-3′	5′-CCGTTCTCTGCCTTGACTGT-3′
YWHAZ	5′-AGACGGAAGGTGCTGAGAAA-3′	5′- CGTTGGGGATCAAGAACTTT-3′
